# Business environment and its impact on high-quality economic development: A configurational analysis of enhancement pathways

**DOI:** 10.1371/journal.pone.0302508

**Published:** 2025-01-13

**Authors:** Guanyu Hou, Chengji Sun, Zishun Lu

**Affiliations:** 1 School of Marxism, Northeastern University, Qinhuangdao, China; 2 School of Business, China University of Political Science and Law, Beijing, China; 3 College of Economics and Management, Southwest University, Chongqing, China; Public Library of Science, UNITED KINGDOM

## Abstract

The nexus between the business environment and high-quality economic development is pivotal for fostering sustainable growth. This study delves into their interrelationship, recognizing its profound practical significance. We have developed a comprehensive index system to evaluate high-quality economic development, encompassing four key dimensions: green development quality, robust economic growth, innovation dynamics, and equitable societal benefits. Concurrently, a business environment index system has been constructed, capturing the nuances of government functionality, social context, and market dynamics. Employing system theory and a configurational approach, this research utilizes the fsQCA method to decode the intricate mechanisms, dynamic pathways, and synergistic interactions among diverse elements within the business environment that influence high-quality economic development. The empirical findings underscore the substantial impact of the business environment on high-quality economic development, highlighting two primary catalyst pathways: the "Rule of Law-Government Efficiency" trajectory and the "Market-Led-Credit Environment" route. These pathways reveal that government efficiency, credit accessibility, legal frameworks, market fairness, and openness are instrumental in driving high-quality economic development. Conversely, inefficiencies in the market mechanism and governmental roles often result in suboptimal development trajectories. The study advocates for policy formulations that recognize and leverage regional distinctiveness, optimizing local resources and strengths to spur high-quality economic development tailored to each region’s unique context.

## I. Introduction

Dedicated to fostering a business environment that is market-centric, anchored in the rule of law, and harmonized with international benchmarks, this approach not only underscores China’s commitment to deepening its open-door policy but also forms a critical foundation for transitioning towards high-quality economic growth. A conducive business environment, as articulated by Jiang Yunchang (2019) [1], adheres to market principles, upholds legal frameworks, and proactively aligns with global business standards. In such a milieu, a transparent market entry mechanism prevails, ensuring free and equitable competition among enterprises and robust protection of property rights. The progressive refinement of core market economic structures–encompassing market entry, property rights safeguards, competitive fairness, and social credit–along with the enhancement of the business milieu, promises to lower corporate operational costs, stimulate entrepreneurial innovation, and attract both domestic and foreign investments. This, in turn, will significantly drive regional economic advancement of high quality and amplify the nation’s overall economic growth trajectory.

Therefore, a thorough analysis of the intrinsic relationship and driving mechanisms between an optimized business environment and high-quality economic development is vital, offering substantial insights for both academic inquiry and policy-making. By elucidating the influence of the business milieu on economic growth, this study provides foundational theoretical support for devising optimization strategies.

The marginal contributions of this study are manifold:

1. From a configurational standpoint, it systematically explores the dynamic driving forces between the business environment and high-quality economic growth, revealing their complex interplay.
2. Utilizing the Fuzzy Set Qualitative Comparative Analysis (fsQCA) method, this research delves into the regional variations in how the business environment catalyzes high-quality economic development, furnishing empirical evidence that enriches our understanding of its impact.
3. This study introduces a comprehensive evaluative index system for both the business environment and high-quality economic growth, laying a theoretical groundwork for future research on how different regions can more effectively foster high-quality economic progress.
4. Finally, through empirical investigations into the developmental status of various regions in China, the study bridges theory with practical application and proposes pragmatic policy recommendations aimed at nurturing a favorable business environment and advancing high-quality economic development.

## II. Literature review and theoretical framework

### (1) Literature review

Since the inaugural publication of the World Bank’s "Doing Business Report," the concept of the business environment has garnered increasing attention from scholars globally. This seminal report conceptualizes the business environment as a composite of external factors encountered by enterprises and market entities across various operational stages, including the establishment of companies and their routine activities. This definition illuminates the multifaceted nature of the business environment, encompassing administrative frameworks, legal systems, economic contexts, and market conditions that entities navigate during market economic operations.

The sophistication of a nation’s business environment is not only a pivotal gauge of its economic soft power but also a critical measure of its overall competitiveness. Scholars, including Li Zhijun (2019) [2], have further elaborated on this notion, characterizing the business environment as a regional "ecosystem." Within this ecosystem, the activities of enterprises are influenced by an array of elements such as governmental institutions, financial bodies, public utilities, and more. This environment spans multiple facets like administration, market dynamics, legal frameworks, and cultural norms, collectively shaping the objective conditions that underpin high-quality economic development. These conditions encapsulate crucial aspects like the clarity of economic policies, the robustness of factor supplies, the efficiency of government services, the integrity of legal systems, the fluidity of resource factors, market fairness, and uniformity in market entry protocols.

Notably, the World Bank provides scores and rankings for the business environments of various countries, offering detailed sub-scores for ten critical areas including taxation, cross-border trade, business initiation, credit accessibility, electricity procurement, and workforce employment. As per the "Doing Business Report 2020," China has witnessed a marked advancement in its business environment, ascending to the 31st position globally in 2020, a notable leap from its standing in 2019. This achievement underscores the country’s strides in refining its business milieu, aligning with global standards and facilitating economic activities more effectively.

China is currently transitioning from a phase of rapid growth to a new era of high-quality economic development. In line with contemporary development paradigms, high-quality economic development is characterized by "innovation, coordination, green, openness, and sharing." This multifaceted approach holistically encapsulates a nation’s comprehensive economic development status. The academic consensus is that high-quality economic development epitomizes the new development philosophy, positioning innovation as the primary impetus, integration as an intrinsic quality, environmental sustainability as a prevailing mode, openness as a necessary path, and inclusivity as the ultimate goal of development. Empirical studies, including those by Zhang Jigang et al. (2022) [3], have illustrated the critical role of a favorable business environment in catalyzing high-quality economic growth. An optimized business environment significantly boosts total factor productivity, thereby propelling high-quality economic growth via fostering bidirectional investment and corporate innovation.

The impact of the business environment on high-quality development is profound and multifaceted. Globally, the effects of the business environment on high-quality economic growth vary across developed and developing countries, revealing heterogeneities in outcomes. Understanding how the various elements within the business environment synergistically influence high-quality economic development is crucial for both academic and practical purposes. Current scholarly focus on the role of the business environment in high-quality economic development encompasses several areas:

1. Direct Impact on High-Quality Economic Development: Economists, including Acemoglu & Robinson (2012) [4–6] and **Porter (2006)** [7], assert that a conducive business environment significantly enhances economic growth, primarily by improving total factor productivity. This environment encompasses both tangible aspects like infrastructure and policies, and intangible elements such as institutional, cultural, and social factors (North, 1990) [8]. A fair, transparent, and stable business environment encourages investment and strengthens economic ties both domestically and internationally (Peng, 2003) [9], offering a competitive advantage in the global economy (Rodrik et al., 2004) [10].
2. Indirect Impact on High-Quality Economic Development: A positive business environment is instrumental in stimulating corporate innovation, a key driver of high-quality economic growth (Schumpeter, 1934) [11]. Innovation leads to technological advancement, quality enhancement of products and services, and can trigger industrial transformation (Romer, 1990) [12]. Additionally, a supportive business environment encourages corporate social responsibility (Porter & Kramer, 2006) [7, 13], which has become integral to sustainable development and can enhance public trust and reduce operational risks (Friedman, 2007) [14].
3. Heterogeneity in Impact: The impact of the business environment on economic development can vary significantly across regions, even under a uniform national policy (Rodríguez-Pose, 2013) [15]. These differences stem from regional disparities in resources, governance, industrial structures, and human capital (Glaeser, 2012) [16]. Local government policies also play a crucial role in shaping the business environment (Enikolopov, 2014) [17]. Therefore, constructing a high-quality business environment involves optimizing local resource allocation, catering to market demands, and leveraging policy support from local governments.

To encapsulate, the current scope of research in this field exhibits certain limitations that warrant attention:

1. Focus on Singular Quantitative Analysis: Predominantly, existing studies have centered on quantitative analyses of individual components of the business environment, often neglecting the complex interactions and collective impacts within the system. This approach may lead to a superficial grasp of the holistic, synergistic, and dynamic aspects of the business environment.
2. Linear Relationship Emphasis: The bulk of research has tended to emphasize the linear relationship between the business environment and economic growth. There is a notable scarcity of studies adopting a configurational perspective to dissect the influence of the business environment on high-quality economic development. This gap in research methodology may lead to an oversimplified understanding of the diverse driving forces and their interplay in fostering high-quality economic development.
3. Lack of Focus on Regional Disparities: The existing body of literature reveals a significant gap in examining regional variations in the role of the business environment in propelling high-quality economic growth. Considering the marked differences in business environments across various regions, it is crucial to explore how to optimally utilize and capitalize on the unique attributes and strengths of each region to advance high-quality economic development. This understanding is not only academically valuable but also holds substantial practical relevance in policy formulation and regional economic planning.

### (2) Theoretical framework

While existing studies have laid a solid foundation in exploring the business environment and high-quality economic development, there remains a gap in research, particularly from a configurational perspective, focusing on specific regions in China as case studies to analyze the concrete pathways through which the business environment influences high-quality economic development. This study aims to fill this gap by theorizing and examining the constitutive elements of the business environment and their impact on high-quality economic development, as follows:

1. Indicators Related to Government Role: Research has established the significance of government involvement, capabilities, and public resource provision in influencing economic growth quality. Government engagement is evident in policy stability, fairness, and transparency, which are critical in shaping business decision-making and confidence, thus impacting economic growth quality (North, 1990) [8]. Government capability is reflected in administrative efficiency, scientific policy formulation, and resource allocation. Effective governments can optimally distribute resources, steering enterprises towards efficiency, environmental sustainability, and social justi**ce (Acemoglu and Johnson, 2005)** [18]. Public resource provisioning, including infrastructure, education, and healthcare, directly affects enterprise efficiency and worker productivity, influencing economic growth quality (Romer, 1986) [19]. Governmental policies and investments significantly shape the economic environment, with well-crafted policies and investments enhancing economic quality (Alesina et al., 2005) [20].
2. Indicators Related to Social Environment: The role of social trust and the rule of law in high-quality economic development is pronounced. The stability and fairness of the social environment are crucial for economic growth. Social trust, encompassing integrity, business ethics, and credit systems, can reduce transaction costs and boost market confidence, thus optimizing resource allocation and enhancing economic growth quality (Zak and Knack, 2001; Guiso et al., 2004) [21, 22]. The rule of law, in its comprehensiveness and fairness, ensures fair competition and fosters innovation, driving high-quality economic development (Acemoglu and Johnson, 2005; Knack & Keefer, 1997) [18, 23].
3. Indicators Related to Market Environment: Factors like market competition fairness and openness significantly impact high-quality economic development. The market environment’s competitiveness and openness influence business strategies and investment decisions, affecting economic growth quality and efficiency (Melitz & Trefler, 2012) [24]. A fair and open market stimulates innovation and enhances product and service quality, thus improving economic growth quality (Aghion et al., 2005; Sondermann D, 2014) [25, 26].
4. Core Indicators of High-Quality Economic Development: These include green development quality, economic growth quality, innovative development quality, and shared public welfare quality, all of which are intricately linked to the business environment. A fair and open market ignites entrepreneurial innovation, enhancing economic innovative quality (Aghion et al., 2005) [27]. Government involvement and public resource provision improve shared public welfare quality (Besley & Ghatak, 2007) [28]. Government optimization of policies promotes green development, thus elevating overall economic growth quality (Barbier, 2010) [29].

**Fig 1:**
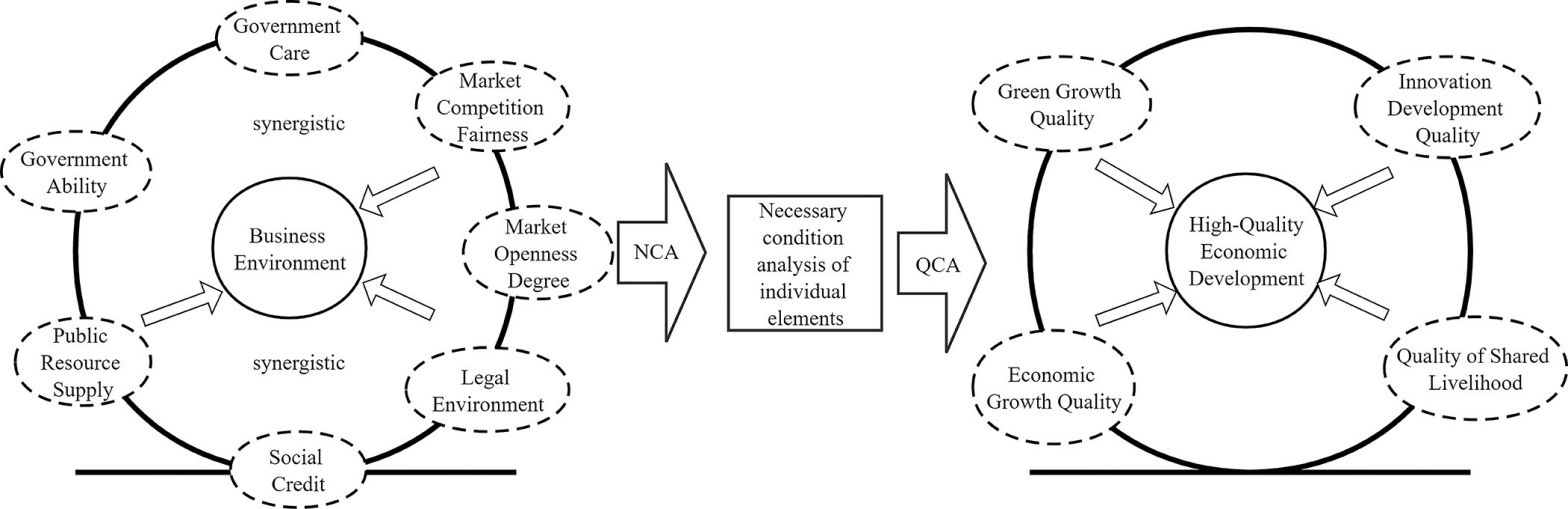
Theoretical framework of the business environment promoting high-quality economic development.

Consequently, this study develops an indicator system for high-quality economic development across four dimensions: green development quality, economic growth quality, innovative development quality, and shared public welfare quality. The business environment is conceptualized through primary indicators concerning government role, social environment, and market environment. These are further delineated into secondary indicators across seven dimensions: government care, governmental capability, public resource provisioning, social trust, rule of law environment, market competition fairness, and market openness. Recognizing the complex and non-linear relationships among these elements, the study proposes a theoretical model from a configurational perspective, as depicted in Fig 1. This approach acknowledges the symbiosis, competition, and evolution of each element and their non-linear impact on high-quality economic development.

## III. Research methodology

### (1) Method selection

Qualitative Comparative Analysis (QCA) represents an innovative research methodology that bridges the gap between case-oriented qualitative approaches and variable-oriented quantitative analyses (Ragin, 2014) [30]. Developed by American sociologist Charles Ragin in 2000 (Ragin, 2000) [31], QCA aims to uncover intricate causal relationships through a unique blend of set theory and Boolean algebra. It focuses on how different constellations of antecedent conditions contribute to varying outcomes. Unlike traditional analytical methods such as the Logit model or cross-tabulation tests, which typically categorize outcome variables, QCA translates variables into sets, thus facilitating more nuanced and comparative analyses between cases.

QCA is categorized into three distinct types based on the configuration of sets: crisp-set QCA (csQCA), fuzzy-set QCA (fsQCA), and multi-value QCA (mvQCA). This study utilizes the fsQCA approach for several compelling reasons:

1. Theory-Practice Integration: FsQCA is exemplary in blending theoretical constructs with practical data. It converts both antecedent factors and outcome variables into set data, offering a more sophisticated analysis than csQCA and mvQCA. This method is distinguished by its capacity to calibrate variables into degrees of membership, represented as fuzzy set values that range continuously within the interval [0,1].
2. Applicability to Continuous Variables: The variables concerning the business environment and high-quality economic development across Chinese provinces are inherently continuous. The fsQCA’s approach to coding these continuous variables aligns perfectly with the study’s needs. It adeptly handles the complexities of concurrency, non-symmetry, and equifinality, which are often challenges in traditional research methodologies. This alignment makes fsQCA particularly suited to the empirical conditions of this research.
3. Insightful Analysis of Complex Relationships: By employing fsQCA, this study delves into the varied experiences and influences of the business environment on high-quality economic development. It provides a novel perspective for exploring and understanding the complex "black box" of causal relationships. Moreover, the application of fsQCA offers substantial theoretical support for crafting regional strategies aimed at fostering high-quality development. This methodological choice thereby enriches the study’s analytical depth and contributes to a more nuanced understanding of the dynamic interplay between the business environment and economic development.

### (2) Sample and data

In this study, the sample encompasses 30 provinces, municipalities, and autonomous regions across mainland China. An index system for high-quality economic development has been established, encompassing four dimensions: quality of green development, quality of economic growth, quality of innovation development, and quality of public welfare sharing. Similarly, a comprehensive business environment index system has been constructed, covering seven aspects: governmental attention, governmental efficiency, public resource provision, financing and credit availability, legal framework development, market fairness, and openness of the market. The data for this research is meticulously sourced from a variety of authoritative publications and databases, including the "China Statistical Yearbook," "China City Statistical Yearbook," "China Business Report," "China Intellectual Property Yearbook," "China Government-Business Relations Report," "China Provincial and Municipal Government Electronic Service Capability Index Report," "China Regional Innovation Capability Evaluation Report," "China Trade and Economic Statistics Yearbook," "China Outward Direct Investment Statistical Bulletin," and the WIND database.

Recognizing the time-lagged impact of the business environment on high-quality economic development, and to mitigate the issue of data singularity, this study follows the practices of scholars like Zhang et al. (2020) [32]. Accordingly, the variables from the year 2020 are paired with the average values of the condition variables from the preceding year. This methodology ensures a more accurate and meaningful analysis of the dynamic relationship between the business environment and economic development. The detailed construction of the index system and the rationale behind the selection of these dimensions and variables are meticulously outlined in Table 1 below. This comprehensive approach provides a robust framework for assessing the nuanced influences of the business environment on the multifaceted aspects of high-quality economic development in China (illustrated in Table 2).

**Table 1:** Conditional variable indicator system.

First-Level Indicator	Second-Level Indicator	Specific Content & Calculation Formula
Government Role	Government Care	Government Care for Enterprises Index
	Government Ability	E-governance Level (Electronic Service Capability Index) Government Expenditure Scale (General Public Budget Expenditure / GDP)
Social Environment	Public Resource Supply	Water, Electricity, Transportation, Human Capital, Average Internet Usage per Person
	Social Credit	Social Financing Environment (Increment of Regional Social Financing Scale / GDP) Social Enterprise Credit (Business Disputes / Number of Enterprises)
	Legal Environment	Judicial Fairness (Judicial Civilization Index), Property Rights Protection (Number of Administrative Rulings on Patent Infringement Disputes / Number of Patents), Social Security (Number of Criminal Cases per 10,000 People), Judicial Service (Number of Lawyers / Number of Enterprises), and Judicial Transparency (China Judicial Transparency Index)
Market Environment	Market Competition Fairness	Includes Establishment of Enterprise Brands (Number of Trademark Registrations / Population) Number of Newly Established Start-up Enterprises, Proportion of Non-public Economy (Number of Private Enterprise Legal Entities / Number of Enterprise Legal Entities)
	Market Openness Degree	Trade Dependence (Customs Import and Export Value as a Percentage of GDP) Foreign-funded Enterprises Ratio (Number of Foreign-funded Enterprises / Number of Enterprises)

Note: Based on multiple calculations using QCA, the author has excluded other factors that are consistent across different regions and have no impact, and did not incorporate them into the indicator system.

**Table 2:** Result variable indicator system.

Primary Indicators	Secondary Indicators	Specific content and calculation formula
Green Growth Quality	Energy consumption per unit of GDP	Total energy consumption (tons of standard coal) / GDP (ten thousand yuan)
	Rate of decrease in energy consumption per unit of GDP%	(Last year’s energy consumption per unit of GDP—this year’s energy consumption per unit of GDP) / last year’s energy consumption per unit of GDP
Economic Growth Quality	Per capita GDP growth rate%	(This year’s per capita GDP—last year’s per capita GDP) / last year’s per capita GDP
	Capital productivity%	GDP / total fixed asset investment in society
	Labor productivity%	GDP / number of employed personnel
Innovation Development Quality	Intensity of R&D fund investment%	R&D/GDP
	Scientific and technological innovation results	Number of invention patents per 10,000 people
Quality of Shared Livelihood	Urban-rural income ratio%	Urban residents’ per capita disposable income / rural residents’ per capita disposable income
	Intensity of education investment%	Education expenditure / budget expenditure
	Intensity of social security service investment	Social security and employment expenditure / budget expenditure

### (3) Measurement and calibration

In the realm of Qualitative Comparative Analysis (QCA), calibration is a critical step that translates quantitative data into qualitative insights. This process is implemented through two primary methods: binary calibration and fuzzy set-based calibration. Binary calibration employs a dichotomous approach, where 0 signifies ’complete non-membership’ in the set, and 1 indicates ’full membership.’ Conversely, fuzzy set-based calibration provides a more nuanced approach, establishing a continuum between 0 and 1 to represent varying degrees of set membership. This method effectively captures the qualitative shift from quantitative data, reflecting the ’effectiveness’ or relevance of each observation in relation to the set.

In operationalizing the fsQCA method, the study begins by segmenting the value ranges of each condition variable and outcome variable based on the actual economic development profiles of the regions under study. This segmentation involves identifying three pivotal ’anchor points’: ’complete belonging,’ ’midpoint’ (or ’cross-point’), and ’complete non-belonging.’ Each condition and outcome variable is then calibrated using the 95th, 50th, and 5th percentiles of the sample data, respectively. This calibration approach aligns with the actual distribution of the sample data, allowing for a more precise determination of the value range and membership degree of each variable.

The outcome of this calibration process is meticulously detailed, culminating in a comprehensive and coherent data table (illustrated in Table 3). This methodological approach is instrumental in unraveling the causal linkages between the business environment and high-quality economic development. Moreover, it provides valuable insights for further theoretical exploration and practical policy-making. By translating quantitative measurements into qualitative assessments, this study offers a more profound understanding of the complex interactions within the business environment and their implications for fostering high-quality economic development.

**Table 3:** Calibration of result variables and condition variables.

Variable Category	Variable Name	Variable Description		Full Membership	Crossing Point	Non-membership
Result Variable	Level of High-Quality Economic Development	*Y*	Level of High-Quality Economic Development	114	95	68
Condition Variables	Government Role	*X1*	Government Care	89	52.	14
		*X2*	Government Ability	90	72	40
	Social Environment	*X3*	Public Resource Supply	66	49	33
		*X4*	Social Credit	87	66	38
		*X5*	Legal Environment	64	43	22
	Market Environment	*X6*	Market Competition Fairness	64	38	19
		*X7*	Market Openness Degree	49	9	2

Note: The high-quality economic development level indicator is processed after calibration using the entropy method.

## IV. Empirical analysis

### (1) Necessity analysis of individual conditions

To elucidate the integral relationships within our study, we conducted an analysis of the necessity of individual conditions. This involved examining whether each condition variable has a necessary relationship with the outcome variable. In fsQCA, condition variables exhibiting a consistency level above 90% are typically recognized as necessary conditions for the outcome. Essentially, this implies that the presence of such a condition or a combination thereof is imperative for the occurrence of the designated outcome.

In our investigation, we assessed whether each individual variable, in relation to the outcome variable, could be deemed as either a sufficient or necessary condition. This assessment was approached from two angles: consistency and coverage. Consistency measures how uniformly a specific result occurs in tandem with a condition variable across all cases. Coverage, on the other hand, gauges the significance of that condition variable among all potential conditions that could lead to a particular result (Ragin, 2009) [33]. The analysis was carried out using the fsQCA 3.0 software, and the non-presence of single variables (denoted by the "~" symbol) was similarly scrutinized.

The findings of this analysis, detailed in Table 4, reveal that the consistency indices of all single variables fall short of the 0.9 threshold necessary to establish a necessary condition. This outcome suggests that no individual variable can independently act as a necessary condition for fostering high-quality economic development. In other words, a solitary variable lacks the capacity to comprehensively account for the nuances and complexities of high-quality economic development. This insight underscores the multifaceted and interdependent nature of the factors that drive high-quality economic development, highlighting the importance of considering a broader range of variables and their interactions in our analysis.

**Table 4:** Necessity test results of single conditions.

Variables	High Level of High-Quality Economic Development		Non-High Level of High-Quality Economic Development	
	Consistency	Coverage	Consistency	Coverage
X1	0.745806	0.806137	0.572414	0.578801
~X1	0.610323	0.604087	0.808276	0.748403
X2	0.770968	0.835664	0.503448	0.510490
~X2	0.548387	0.541401	0.837931	0.773885
X3	0.684516	0.731220	0.544138	0.543763
~X3	0.572903	0.573273	0.731034	0.684312
X4	0.718065	0.761807	0.606207	0.601643
~X4	0.624516	0.628980	0.760000	0.716049
X5	0.709677	0.734312	0.520690	0.504005
~X5	0.520645	0.537284	0.725517	0.700400
X6	0.806452	0.845738	0.493793	0.484438
~X6	0.508387	0.517740	0.842759	0.802891
X7	0.728387	0.785118	0.531724	0.536161
~X7	0.569677	0.565301	0.786897	0.730474

### (2) Sufficiency analysis of condition configurations

Building upon the analysis of the necessity of single variables, the study then progresses to a configurational sufficiency analysis. This analysis aims to discern how various combinations or configurations of multiple conditions can culminate in certain outcomes. In this context, if we consider conditions and outcomes as distinct sets, the analysis of multi-condition configurations seeks to determine whether the set of conditions constitutes a subset of the outcome set. Consistency remains the key metric for assessing the sufficiency of these configurations. Generally, for a configuration to be considered sufficiently reliable in leading to an outcome, its consistency level should not fall below 0.75. The choice of a frequency threshold, which is pivotal for identifying relevant configurations, is contingent upon the sample size. For small to medium-sized samples, a frequency threshold of 1 is considered adequate, while for larger samples, this threshold should be set above 1.

Through the lens of configurational analysis, this study identified key pathways by which the business environment influences high-quality economic development. As delineated in Table 5, the impact of the business environment on high-quality economic development predominantly manifests through five pathways. These pathways incorporate critical factors such as governmental efficiency, the credit environment, the rule of law environment, and the market’s fairness and openness. Different combinations of these factors, in their respective configurations, collectively facilitate the achievement of high-quality economic development. The analysis yielded a total coverage of 56%, with an overarching consistency of 97%. Notably, the consistency of each individual pathway, as well as the combined consistency, exceed the 0.97 mark. This high level of consistency underscores the significance of these five pathways as essential strategies for leveraging the current business environment to bolster high-quality economic development. Such findings highlight the complex interplay of various factors within the business environment and their combined potential in shaping the trajectory of economic development towards higher quality and sustainability.

**Table 5:** Configuration of high (Non-high) quality economic development.

Condition Variables		High Level of High-Quality Economic Development					Non-High Level of High-Quality Economic Development			
		H1	H2	H3	H4	H5	L1	L2	L3	L4
Government Role	X1		☒	●	●	●	◎	◎	◎	◎
	X2	★	★	●	●		◎	◎	◎	◎
Social Environment	X3	●	●	●		●	●	☒	☒	☒
	X4	☒	●	★	★	★	☒	●	☒	☒
	X5	★	★		●	●	☒	☒	★	★
Market Environment	X6	●	●	★	★	★	◎	◎	◎	◎
	X7	☒		★	★	★	◎	◎	◎	◎
Consistency		0.977	0.990	1	0.994	0.982	0.919	0.941	0.909	0.920
Raw Coverage		0.274	0.270	0.370	0.381	0.354	0.297	0.312	0.291	0.246
Unique Coverage		0.038	0.003	0.058	0.070	0.042	0.028	0.090	0.053	0.024
Total Coverage		0.561					0.508			
Overall Consistency		0.974					0.931			

Note: The symbol ★ represents core conditions, ● represents peripheral conditions, ◎ represents absence of core conditions, ☒ represents absence of peripheral conditions, and a blank space indicates that the condition can either be present or absent, without affecting the accuracy of the path. The same applies below.

#### 1. Analysis of high configuration results

Configuration H1 exhibits a high consistency of 0.977 and an original coverage of 0.274, representing 27% of the sample cases. The predominant factors are government efficiency and the rule of law environment, complemented by public resource provision and market fairness. For instance, Anhui Province exemplifies this configuration by rigorously fortifying laws and regulations, ensuring judicial fairness, and safeguarding the legal rights of enterprises and individuals. Such efforts in creating a transparent business environment have effectively attracted investments, thereby catalyzing high-quality economic development.

Configuration H2, with a consistency of 0.990 and an original coverage of 0.270, accounts for another 27% of the cases. It shares core factors with H1—government efficiency and the rule of law environment—while adding public resource provision, credit environment, and market fairness as auxiliary factors. Jiangsu Province is a case in point, where it emphasizes market fairness and government efficiency by intensifying market supervision, reforming administrative systems, and fostering fair competition, thereby facilitating high-quality economic development.

Configuration H3 achieves a perfect consistency of 1 and covers 0.37 of the cases (37%). Its key factors are the credit environment, market fairness, and market openness, supported by government care, efficiency, and public resource provision. Zhejiang Province, for example, has focused on market fairness and openness, implementing measures to enhance market regulation, attract foreign investment, and participate in international economic cooperation, thereby advancing high-quality economic development.

Configuration H4, with a consistency of 0.994 and covering 38% of the cases, highlights the credit environment, market fairness, and openness as core factors. It is further supported by government care, efficiency, and the rule of law environment. Beijing demonstrates this pathway by promoting market fairness, openness, and technological innovation, enhancing cooperation among various institutions, and providing support for innovative enterprises.

Configuration H5, demonstrating a consistency of 0.982 and covering 35% of the sample, focuses on the credit environment, market fairness, and openness as central factors, with government care and market resource supply as auxiliary elements. Shandong Province’s commitment to improving the credit system, enhancing financial services, and supporting small and medium-sized enterprises exemplifies this configuration.

Overall, these five pathways (H1—H5) reveal regional variations in promoting high-quality economic development, influenced by factors like economic development level, industrial structure, and resource endowment. Despite these differences, a common focus across regions is on enhancing market fairness and openness to stimulate economic vitality and innovation.

It’s noteworthy that regional differences are also apparent in the social environment and government role. Some areas prioritize government efficiency and rule of law to optimize governance and services, while others focus more on improving the credit environment and market resource supply to protect legal rights. These variations underscore the distinct characteristics and priorities of each region in fostering high-quality economic development, offering a nuanced understanding of the diverse strategies and pathways employed across different regions.

#### 2. Analysis of low configuration results

The study identifies four distinct low configurations, collectively exhibiting a consistency of 0.931 and covering 50.8% of the sample. A critical observation from these configurations is that shortcomings in the market environment and government function primarily contribute to their formation. However, this does not imply that regions with low configurations have inferior business environments or lesser degrees of high-quality economic development. Rather, these configurations highlight areas where the business environment’s influence on high-quality economic development is not fully realized, indicating the presence of specific obstacles and challenges.

Configuration L1, typified by Gansu Province, shows a consistency of 0.919, with unique coverage at 0.028 and original coverage at 0.297. This configuration exemplifies a scenario where the business environment’s role in driving high-quality economic development is less pronounced. Qinghai Province, representing Configuration L2, demonstrates a consistency of 0.941, unique coverage of 0.090, and original coverage of 0.312, marking another low configuration case. Jilin Province, the representative for Configuration L3, has consistency, unique coverage, and original coverage figures of 0.909, 0.053, and 0.291, respectively. It signifies the third low configuration of the business environment’s role in fostering high-quality economic development. Lastly, Configuration L4, represented by the Xinjiang Uygur Autonomous Region, records a consistency of 0.920, unique coverage of 0.024, and original coverage of 0.246, being the fourth instance of a low configuration.

For these low-configuration regions to enhance the business environment’s impact on high-quality economic development, improvements are necessary, particularly in market environment and governmental roles. Steps such as strengthening market regulation, refining government services, and enhancing the quality of the legal environment are crucial. Through these measures, these regions can significantly improve their business environments, thereby effectively propelling themselves towards the goal of high-quality economic development. This analysis underscores the need for tailored strategies that address specific regional challenges and leverage unique opportunities to foster a more conducive business environment for sustainable economic progress.

#### 3. Summary analysis of high and low configuration paths

When comparing the high and low configurations of business environments driving high-quality economic development, it becomes apparent that the low configuration is not simply the antithesis of the high configuration. Instead, the low configuration underscores areas where the business environment’s influence on high-quality economic development is less pronounced, pointing to potential deficiencies and areas necessitating reinforcement. This distinction underscores the asymmetry in the prerequisites for leveraging the business environment to foster high-quality economic development.

From the analysis of the five high configurations, two primary driving pathways emerge. The first pathway is the "Rule of Law—Government Efficiency" driven type (H1-H2), emphasizing the pivotal role of legal frameworks and governmental efficiency in advancing high-quality economic development. The second pathway, the "Market-led—Credit Environment" driven type (H3-H5), focuses on the influential role of market mechanisms and the credit environment in driving high-quality economic development. These pathways offer distinct strategic options for local governments and businesses, aiding in the effective utilization of business environment advantages to achieve high-quality economic development goals.

"Rule of Law—Government Efficiency" Driven Type Analysis (H1-H2):

This pathway highlights the essential role of the rule of law and government efficiency. Regions following this path, like Anhui province, have made significant strides in legal system development and government efficiency enhancement. Anhui has pioneered innovative platforms and supported financial risk management strategies to ensure a stable and conducive business environment. Similarly, Jiangsu province’s comprehensive promotion of the "five environments" strategy, including policy, market, government affairs, rule of law, and humanities, exemplifies this pathway. The province’s multifaceted approach to improving consumer market competitiveness and creating a distinctive market environment with Jiangsu characteristics has effectively propelled its high-quality economic development.

"Market-led—Credit Environment" Driven Type Analysis (H3-H5):

This pathway underscores the central role of market environment factors and the importance of market fairness, openness, and a robust credit environment. Regions like Zhejiang province have implemented targeted policies to expand investment, support technological innovation, and promote advanced manufacturing, all geared towards high-quality economic development. Beijing’s unique approach, focusing on quality improvement and steady scale growth, leverages the "five-child" linkage model to foster a world-class science and technology park and promote high-quality development in its sub-city center. Shandong province, through its "Stable Progress" policy, aims to cultivate market demand, emphasizing green, low-carbon development, and the advancement of its manufacturing sector.

These analyses reveal that different regions employ varied strategies to harness their unique business environments for high-quality economic development. The contrasts between the high and low configuration paths offer valuable insights into the diverse approaches and potential areas for improvement in leveraging the business environment effectively across different regional contexts.

### (3) Analysis of configuration substitution relationship

The comparative analysis of the high configurations suggests the presence of overlapping and substitution relationships among the elements within the paths. By examining the original and unique coverage of each configuration, we identify potential substitutions among the elements, leading to an in-depth comparative analysis of the high-level paths.

#### 1. Comparative analysis of Path H1 and Path H2

Both Path H1 and Path H2 are categorized under the "Rule of Law—Government Efficiency" driven type, yet they exhibit distinct approaches to achieving high-quality economic development. Path H1 places greater emphasis on the construction of the rule of law environment and the government’s lead in economic development, highlighting significant governmental accomplishments in legal system building and efficiency enhancement. Conversely, Path H2 leans more towards improving government efficiency and fostering synergy between government and market, underscoring the active role of the government in a rule-of-law environment to co-drive high-quality economic development with the market.

Comparing Path H1 and Path H2 reveals a degree of substitutability in promoting high-quality economic development. For instance, in regions with a well-established rule of law environment, enhancing government efficiency may more directly spur high-quality economic development. Conversely, in areas with high government efficiency, strengthening the rule of law environment may profoundly influence economic growth. Thus, under varying regional conditions, Path H1 and Path H2 can complement and collectively advance high-quality economic development.

#### 2. Comparative analysis of Paths H3, H4, and H5

Paths H3, H4, and H5 fall under the "Market-led—Credit Environment" driven type, each with unique approaches to actualizing high-quality economic development. Path H3 accentuates the dominant role of market environment factors, particularly market fairness and openness. Path H4 concentrates on developing the credit environment, positing that a robust credit system environment is a key driver of high-quality economic development. Path H5, meanwhile, focuses on the interplay between the market and credit environments, emphasizing their joint positive impact on economic development.

The comparison of Paths H3, H4, and H5 indicates substitutability and complementarity in fostering high-quality economic development. Optimizing market environment factors, for example, may enhance the credit environment, and a well-functioning credit environment can further bolster the market. Regions can select appropriate paths based on their specific conditions, learning from each other’s strengths and experiences. Integrating market and credit environment advantages can achieve multi-path complementarity, jointly propelling high-quality economic development.

In conclusion, this comparative analysis of different high-configuration paths elucidates the critical roles and interplay of the rule of law environment, government efficiency, market environment, and credit environment in driving high-quality economic development. It deepens the understanding of the specific needs and comparative advantages of various regions in pursuing economic growth, offering valuable insights for tailored policy formulation to support regional economic objectives.

### (4) Spatial contextual differences of business environment’s impact on high-quality economic development

The impact of the business environment on high-quality economic development varies across China’s eastern, central, and western regions due to distinct differences in factor endowment, economic development levels, openness, and institutional environments. Accordingly, these regions are categorized as per national standards, with the three northeastern provinces included in the eastern region. For a more nuanced analysis, the 95th percentile, 50th percentile, and 5th percentile are redefined as anchor points representing full membership, crossover, and non-membership, respectively. This recalibration allows for a detailed comparison and analysis of the business environment’s role in fostering high-quality economic development across these regions, with the findings presented in Table 6.

**Table 6:** Configurational analysis of high-quality economic development (Eastern, central, and western regions).

Condition Variables		High Level of High-Quality Economic Development						
		E1	E2	E3	M1	M2	W1	W2
Government Role	X1	●	●		☒	●	★	★
	X2	●		●	●	☒	★	★
Social Environment	X3	★	★	★	●	☒	●	
	X4	●	●	●	●	☒	☒	●
	X5		●	●	★	★	★	★
Market Environment	X6	●	●	●		●	★	★
	X7	●	●	●	☒	☒	☒	●
Consistency		1	1	0.992	1	1	0.965	1
Raw Coverage		0.376	0.391	0.356	0.390	0.277	0.526	0.420
Unique Coverage		0.063	0.078	0.043	0.175	0.062	0.137	0.031
Total Coverage		0.499			0.420		0.557	
Overall Consistency		0.994			1		0.967	

The analysis reveals three prominent high-configuration pathways in the eastern region (E1-E3). These pathways suggest that a robust supply of public resources, coupled with a favorable credit environment and a fair, open market, can significantly enhance the level of high-quality economic development in the eastern region. In contrast, the central region (M1-M2) demonstrates that the rule of law environment is a pivotal factor in promoting high-quality economic development. Here, the government’s role and the market environment can somewhat mitigate the challenges posed by a lack of mutual conditions during development.

In the western region (W1-W2), the analysis indicates that governmental influence can offset constraints imposed by factors like the credit environment and market openness, thereby facilitating high-quality economic development. This suggests that the western region’s pathway to high-quality economic development is more reliant on effective government intervention and support.

Overall, these results highlight the spatial contextual differences in how the business environment influences high-quality economic development across China’s diverse regions. Understanding these variations is crucial for formulating region-specific strategies that leverage local strengths and address unique challenges. This spatially differentiated approach is essential for achieving balanced and inclusive economic development across the country.

The distinctions in business environment trajectories fostering high-quality economic growth across China’s eastern, central, and western regions can be attributed to several practical factors:

1. Factor Endowments Variability: There is a pronounced disparity in natural resources, human capital, technological proficiency, and infrastructural development across these regions. The eastern region boasts a robust economic base, advanced technological capabilities, and a substantial talent pool. In contrast, the central and western regions exhibit developmental delays in these areas. These variances influence each region’s developmental focus and policy-making decisions.
2. Economic Development Divergence: The eastern region is characterized by a higher economic development level, underpinned by a strong industrial foundation and vibrant market dynamics. The central region is in a transitional phase, actively restructuring and optimizing its industrial framework. Although the western region is experiencing rapid growth, its overall economic magnitude and development stage lag behind. Consequently, each region faces unique challenges, opportunities, and approaches in pursuing high-quality economic advancement.
3. Openness Level Disparities: Geographical positioning and historical factors contribute to the eastern region’s heightened openness. Here, foreign trade and investment are pivotal. Conversely, the central and western regions demonstrate lower openness levels, necessitating enhancement in international competitiveness. These disparities dictate each region’s specific focus and strategic path in pursuing high-quality economic progress.
4. Institutional Environment Differences: Historical and geographical influences have led to notable variations in institutional environments across regions, encompassing aspects like legal frameworks, government administration, and market oversight. The eastern region enjoys a more mature rule of law and government governance, while the central and western regions present opportunities for further development in these areas. These differences necessitate region-specific attention to distinct facets of challenges in promoting a conducive business environment for high-quality economic growth.

In summary, the divergent paths of business environments conducive to high-quality economic development in China’s eastern, central, and western regions originate from disparities in factor endowments, economic development levels, openness, and institutional frameworks. These variances manifest in distinct resource allocations, economic scales, development stages, industrial structures, openness in international trade and investment, and institutional governance. Addressing these unique regional challenges and opportunities is crucial for tailored policy formulation and implementation, aiming to achieve high-quality economic development goals.

Furthermore, for robust spatial contextual difference analysis, the sample cases are bifurcated into eastern and non-eastern regions. The 95th, 50th, and 5th percentiles serve as qualitative anchors for full membership, crossover, and non-membership, respectively. This methodology confirms the relative stability of the spatial differential configuration findings.

### (5) Robustness check

To ensure the validity of our findings, we employed several methods for robustness verification:

1. Adjustment of PRI Threshold: In line with the methodology proposed by Lin Yan and Lu Junyao (2022) [34], we modified the PRI (Possibility Ratio Index) consistency threshold for Qualitative Comparative Analysis (QCA). The threshold was altered from 0.8 to 0.85 and 0.90, respectively. Following this adjustment, the pivotal conditions and the consistency of the identified high-configuration pathways remained aligned with our initial findings. This consistency reinforces the reliability of the earlier discussed outcomes.
2. Lagged High-Quality Economic Development Index: Drawing from the research approach of Sun Hao(2020) [35], we recalibrated the high-quality economic development index to include a one-period lag. This revised index was structured around five secondary indicators, encapsulating innovation, coordination, greenness, openness, and sharing, to define a comprehensive high-quality economic development system. This adjustment allows for the examination of the outcome variable over an extended timeframe, thereby enhancing the depth of our analysis.
3. Reclassification of Condition Variables: We also revisited the classification of our condition variables. Specifically, the variables ’government care’ and ’government efficiency’ were amalgamated into a single factor termed ’government role.’ Similarly, ’market fairness’ and ’market openness’ were merged to form the ’market environment’ factor. This reclassification revealed that the fundamental core element conditions in the high configuration paths are in concordance with the results of our prior empirical analysis. This overlap indicates that the core elements are subsets of the high configuration pathways identified earlier, thereby affirming the robustness of our research conclusions.

These robustness checks are integral in confirming the stability and reliability of our research findings, ensuring that our conclusions are not only consistent but also resilient to variations in analytical approaches and variable definitions.

## V. Research conclusions and practical implications

### (1) Research findings

Utilizing systems theory and a configurational approach, this study has developed an index system for the business environment and high-quality economic development. Through the application of the fuzzy-set Qualitative Comparative Analysis (fsQCA) method, we have delved into the determinants, pathways, and synergistic interplays of various business environment elements on high-quality economic growth. Our key conclusions are as follows:

#### 1. Multifaceted preconditions for economic development

Our analysis reveals that isolated factors such as government involvement, social milieu, and market conditions are insufficient as standalone prerequisites for high-quality economic advancement. Instead, elements like government efficacy, credit environment, adherence to rule of law, and a market characterized by fairness and openness are pivotal in the nexus between the business environment and high-quality economic growth. Notably, shortcomings in the market environment and governmental roles emerge as primary factors contributing to low-configurational outcomes.

#### 2. Non-symmetrical and multifarious driving characteristics

The study identifies distinct driving types for high-quality economic development, including the "Rule of Law-Government Efficiency" and "Market Dominant-Credit Environment" models. Moreover, four lower-configurational driving paths are identified, displaying an asymmetric relationship with their higher-configurational counterparts. A comparative analysis of high and low configurations reveals that the latter is not merely an inverse or a diminished state of the former. Instead, it represents barriers and impediments in the pursuit of high-quality economic development.

#### 3. Regional variability in high-configuration performance

There is a marked disparity across different regions in their performance of the business environment’s influence on high-quality economic development. These variations are primarily attributable to differences in regional element endowments, economic development levels, degrees of openness, and institutional frameworks. For targeted promotion of high-quality economic development, it is imperative for each region to formulate strategies tailored to its unique characteristics and circumstances.

In summary, this research underscores the complexity and multifactorial nature of driving high-quality economic development, highlighting the need for a nuanced understanding of regional specificities and a comprehensive approach that transcends simplistic, one-dimensional strategies.

### (2) Practical recommendations

The study’s findings lead to several practical recommendations for fostering high-quality economic growth:

#### 1. Synergistic policy approach

It’s imperative for policymakers to recognize and leverage the interplay among various elements within the business environment. Crafting policies that not only address individual aspects but also create synergies between them is crucial. This approach is especially relevant when enhancing government efficiency, improving the credit environment, upholding the rule of law, and ensuring market fairness and openness. A holistic policy framework, which integrates these elements effectively, can significantly boost the overall trajectory of high-quality economic development.

#### 2. Region-specific strategies

Tailoring strategies to regional economic profiles and developmental stages is vital. For the eastern region, with its human capital and technological strengths, robust infrastructure, and openness, policies could focus on deepening market reforms and spurring technological innovation. The central region, meanwhile, would benefit from bolstering the rule of law, enhancing government efficiency, and refining its market environment, thereby addressing its unique developmental hurdles. The western region should concentrate on infrastructure development and talent nurturing, coupled with efforts to improve government efficiency. Additionally, fostering international partnerships can be a game-changer, bringing in much-needed resources and investment.

#### 3. Rule of law and government efficiency as cornerstones

Governments at all levels should commit to elevating efficiency and reinforcing the legal framework. This commitment involves reforming civil service evaluation systems, enhancing legal literacy among citizens, and streamlining administrative processes to lower operational burdens for businesses. Such initiatives create a more equitable, transparent, and efficient environment for enterprises. Beyond laying a stable foundation for high-quality economic growth, these measures also vitalize the market by fostering an atmosphere of fairness and transparency.

These recommendations emphasize the need for a nuanced, integrated approach to policy-making, where the focus is not only on individual elements but also on how they interact and reinforce each other. This strategy ensures that the journey towards high-quality economic development is both comprehensive and inclusive, catering to the unique needs and potentials of different regions.

### (3) Future research directions

The findings of this study pave the way for several promising avenues for future research:

#### 1. Comparative cross-regional and cross-national studies

Future research could greatly benefit from adopting a comparative lens, focusing on cross-regional and cross-national analyses. This approach would be instrumental in identifying broader patterns and diverse experiences in economic development. For example, tailored studies examining the business environments of various regions can provide deeper insights into the specific challenges and issues they face on the path to high-quality economic development. Such comparative studies can offer valuable lessons and universal strategies applicable across different contexts.

#### 2. Incorporation of additional business environment variables

To enrich our understanding of the intricate relationship between the business environment and high-quality economic development, future studies should consider integrating more variables. These could include factors like entrepreneurial spirit, talent management policies, and industrial strategies. Expanding the range of variables will not only provide a more comprehensive understanding of the dynamics at play but also offer a broader perspective for developing effective and targeted policy interventions.

#### 3. Methodological expansion and integration

Another critical area for future research is the exploration and application of diverse methodological approaches. There is significant potential in combining quantitative methods, such as structural equation modeling, with qualitative techniques like empirical case studies. This methodological pluralism can bring about a more nuanced and multifaceted understanding of the subject matter. Moreover, it can strengthen the validity and reliability of research outcomes, ensuring that they are well-rounded and robust.

In summary, future research in this field should aim for broader comparative analyses, incorporate a wider array of variables related to the business environment, and embrace a mix of research methodologies. These directions not only promise to deepen our understanding of the complexities involved in achieving high-quality economic development but also contribute to the formulation of more effective and context-specific policies and strategies.

## Supporting information

S1 Data (XLSX)
